# Mutator System Derivatives Isolated from Sugarcane Genome Sequence

**DOI:** 10.1007/s12042-012-9104-y

**Published:** 2012-07-06

**Authors:** M. E. Manetti, M. Rossi, G. M. Q. Cruz, N. L. Saccaro, M. Nakabashi, V. Altebarmakian, M. Rodier-Goud, D. Domingues, A. D’Hont, M. A. Van Sluys

**Affiliations:** 1 Departamento de Botânica-IB-USP, GaTE Lab, Brasil, Rua do Matão, 277, 05508-900 São Paulo, SP Brazil; 2 Centre de Coopération Internationale en Recherche Agronomique pour le Développement (CIRAD), UMR AGAP, Avenue Agropolis, 34398 Montpellier Cedex 5, France

**Keywords:** *Saccharum* spp, Poaceae, Mutator transposon, MUSTANG, Genome, Sugarcane

## Abstract

Mutator-like transposase is the most represented transposon transcript in the sugarcane transcriptome. Phylogenetic reconstructions derived from sequenced transcripts provided evidence that at least four distinct classes exist (I–IV) and that diversification among these classes occurred early in Angiosperms, prior to the divergence of Monocots/Eudicots. The four previously described classes served as probes to select and further sequence six BAC clones from a genomic library of cultivar R570. A total of 579,352 sugarcane base pairs were produced from these “Mutator system” BAC containing regions for further characterization. The analyzed genomic regions confirmed that the predicted structure and organization of the Mutator system in sugarcane is composed of two true transposon lineages, each containing a specific terminal inverted repeat and two transposase lineages considered to be domesticated. Each Mutator transposase class displayed a particular molecular structure supporting lineage specific evolution. MUSTANG, previously described domesticated genes, are located in syntenic regions across Sacharineae and, as expected for a host functional gene, posses the same gene structure as in other Poaceae. Two sequenced BACs correspond to hom(eo)logous locus with specific retrotransposon insertions that discriminate sugarcane haplotypes. The comparative studies presented, add information to the Mutator systems previously identified in the maize and rice genomes by describing lineage specific molecular structure and genomic distribution pattern in the sugarcane genome.

**Electronic supplementary material:**

The online version of this article (doi:10.1007/s12042-012-9104-y) contains supplementary material, which is available to authorized users.

## Introduction

The forces that shape the organization of plant genomes are relevant to eukaryotic evolution. Tolerance to polyploidy is a recurrent event and genome size may vary considerably within closely related species. Under this view, the Poaceae species are rather interesting to compare since genome size can vary over 40 fold (245 Mbp to 25,456 Mbp) and ploidy levels range from basic diploids to decaploids (Arumuganathan and Earle 1991; Paterson et al. 2009). Even with this large genome size and ploidy level variation, syntenic regions are detected which show maintenance of gene order which suggest their ancestral origin and conservation of gene function. The C paradox proposed by Thomas (1971) and reviewed in (Jones and Pasakinskiene 2005) somewhat links plasticity of the genome through its association with organism complexity. Among the genetic elements capable of altering genome structure, transposable elements (TEs), originally described by McClintock (1956), are responsible for plant genome size variation as has been repeatedly demonstrated for plants, particularly in Poaceae (Shirasu et al. 2000; Bennetzen and Ramakrishna 2002; Jannoo et al. 2007; Wicker et al. 2001; Vitte et al. 2007). Recent studies are revealing that not only are TEs powerful mechanisms for genome expansion and retraction (Kalendar et al. 2000; Piegu et al. 2006), they also remodel gene content through the generation of new genes (Kazazian 2004; Cordaux et al. 2006) and provide new regulatory networks for altering gene expression (Kashkush et al. 2003; Muotri et al. 2007; Feschotte 2008). TEs are classically sorted into two groups: transposons and retrotransposons. DNA transposons are elements that mostly propagate through a “cut and paste” mechanism dependent on the presence of a specific transposase protein that recognizes the sub-terminal and terminal portion of the element. The transposase encoded by a particular autonomous element preferentially acts on several related copies that share nucleotide similarity at their ends. Movement of retrotransposons depends on active transcription, which provides the substrate to be reverse transcribed into a DNA copy that is reinserted into the genome to increase its copy number after each propagation cycle. Long terminal repeat (LTR) retrotransposons, the most abundant retroelements in plants, are classified on the basis of polyprotein domain order into two major families: the Ty1/copia-like, and Ty3/gypsy-like elements. The replicative potential of these elements has been associated with genome size variation (Kalendar et al. 2000; Du et al. 2006; Ammiraju et al. 2007; Vitte et al. 2007). Expanding the genome-based knowledge from well-studied model plants such as Arabidopsis, rice and maize is fundamental to reveal the impact of these elements in other plant genomes. Studies on barley (Shirasu et al. 2000) and hexaploid wheat (Devos et al. 2005) support the close association of TEs with genome structure.

The present work focuses on sugarcane, a Poaceae member that is cultivated through clonal cuttings, not sexual seeds, of hybrids selected from crosses between two species having different ploidy levels. Modern cultivars were obtained from crosses between the domesticated sugar storing species *Saccharum officinarum* and the wild non-sugar species *Saccharum spontaneum*, followed by several generations of back-crossing and clonal selection (Grivet and Arruda 2002). Both *Saccharum* species have autopolyploid origin, *S. spontaneum* (*X* = 8) with 2n = 40 to 128, and *S. officinarum* (*X* = 10) with 2n = 80. The corresponding monoploid genome sizes are 760 Mb for *S. spontaneum* and 930 Mb for *S. officinarum* (D’Hont and Glaszman 2001). Thus, modern sugarcane cultivars are highly polyploid and aneuploid, with a chromosome number ranging from 100 to 130, of which 70–80 % comes from *S. officinarum*, 10–20 % from *S. spontaneum*, and few chromosomes are derived from inter-specific recombination. Modern sugarcane monoploid genome size is roughly 1Gb based on previous studies (D’Hont 2005), while its close relatives *Sorghum bicolor* and *Oryza sativa* present 730 Mb and 430 Mb, respectively.

Analysis of the sugarcane transcriptome revealed a diverse collection of transposable elements being expressed, among which Mutator-like transposases and Hopscotch-like retrotransposons were the most abundant TE transcripts (Rossi et al. 2001; Araújo et al. 2005). Phylogenetic reconstructions based on 173 amino acids (aa) of the Mutator-like transposases provided evidence that at least four distinct classes (I–IV) exist, and suggest that diversification of the Mutator system occurred early in the evolution of Angiosperms, prior to the divergence of Monocots/Eudicots (Rossi et al. 2004). Saccaro et al. (2007) performed an in silico study on the rice genome and proposed, based on copy number and structural features such as presence of flanking terminal inverted repeats (TIRs), that Classes I and II correspond to bona fide transposons. These authors reported that not only are the rice Mutator-like transposases flanked by TIRs, but also they capture host DNA surrounded by TIRs. These host genome carriers were originally named Pack-MULES (Jiang et al. 2004). The host DNA surrounded by TIRs also contains a transposase-like domain, previously called Transduplicated-MULEs (Juretic et al. 2005), was also found. Classes III and IV clusters in rice and Arabidopsis correspond to previously described classes called MUSTANGs, which represent domesticated transposases (Cowan et al. 2005). Saccaro et al. (2007) also showed that copy number differs greatly between all four classes and, at least in grasses, there was a class-specific amplification of Class II elements.

The aim of the present study is to investigate through high quality BAC sequencing six genomic regions selected to contain each of the four classes of the sugarcane Mutator-like elements. Among the selected regions, two BACs correspond to hom(oe)logous regions of the sugarcane genome and reveal local rearrangements due to retrotransposon activity. Whenever possible, the corresponding orthologous region from rice and sorghum were analyzed to offer support of the hypothesis that retrotransposons are involved in genome expansion in sugarcane. The domesticated elements (Classes III and IV) reside on syntenic regions among rice, sorghum and sugarcane, while true transposon units have no corresponding orthologous insertions. In situ hybridization experiments with Class II Mutator probe revealed that this element amplified to high numbers in the sugarcane genome. Each transposase class analyzed disclosed a particular molecular structure. Finally, the results presented add information to the Mutator system from Poaceae by providing insights on lineage specific molecular structure and genomic distribution patterns in the sugarcane genome, knowledge previously restricted to maize and rice.

## Results

### BAC Selection and Sequence Analysis

The R570 sugarcane cultivar genomic BAC library (Tomkins et al. 1999) was previously screened with class-specific probes to estimate Mutator-like transposase copy number (Saccaro et al. 2007). Prior to sequencing, a total of 358, 2236, 6 and 34 BAC clones were identified as positives for Class I, II, III and IV, respectively. From these a subset of 12 clones for each Classes I, II and IV (total of 36) plus 6 clones identified for Class III were *Hind*III fingerprinted to ensure there was no redundancy (list in Table S1 supplementary material). Membranes containing the restricted BAC clones and *Hind*III digested genomic DNA from each *S. officinarum* (clone Badila) and *S. spontaneum* (clone Mandalay) were prepared and hybridized with class-specific probes to identify the progenitor origin of each BAC. Sugarcane DNA fragments containing the BAC clones 095F04 and 148J07 correspond to two distinct haplotypes determined by their restriction pattern and subsequent sequence. However, their hybridization profiles were not conclusive regarding the parental origin. Seven BACs were sequenced to near completion at a high base call quality to identify the predicted Mutator-like transposase for each clone.

A total of six BACs were sequenced to completion and these correspond to two Class I BACs (115J16 and 086H20), one Class II BAC (007O13), two BAC clones for Class III that correspond to the same locus (095F04, haplotype A) and (148J07, haplotype B), and one BAC clone for Class IV (249C12). A total of 579,352 bases were produced. Fifteen genes in addition to the five predicted Mutator-like transposases were identified based on their sequence similarity to the previously annotated genomes of sorghum and rice (Table 1). Together the 12 non-mutator system genes plus the two Class III and one Class IV MUSTANG genes matched to corresponding syntenic regions in both sorghum and rice genomes. The five remaining BACs (115J16, 086H20, 249C12, 201A23 and 007O13) corresponded to unrelated genomic regions.

**Table 1:** R570 BAC gene index

Sugarcane BAC	Genes^1^	FS or STOP^2^	Orthologous loci		Transcript^5^
			Sorghum^3^	Rice^4^	
095F04 (130,606 bp) R570 haplotype A	Putative DEAH (Asp-Glu-Ala-His) box polypeptide 35 (DEAD-like helicases superfamily) (rnah)	No	Sb03g002030	LOC_Os01g11370	BQ536744 (stem), CA217625 (seedling), CA217706 (seedling).
	Calcium-binding protein (cbp)	No	Sb03g002020	LOC_Os01g11414	CA300700 (etiolated leaf), TA32438_4547 (leaf).
	Class III MUSTANG (SC-MUGA.2)	No	Sb03g002010	LOC_Os01g41210	CA246160 (inflorescence), DQ115076 (callus).
148J07 (116,781 bp) R570 haplotype B	Putative DEAH (Asp-Glu-Ala-His) box polypeptide 35 (DEAD-like helicases superfamily) (rnah)	No	Sb03g002030	LOC_Os01g11370	BQ536744 (stem), CA217625 (seedling), CA217706 (seedling).
	Calcium-binding protein (cbp)	No	Sb03g002020	LOC_Os01g11414	CA300700 (etiolated leaf), TA32438_4547 (leaf).
	Class III MUSTANG (SC-MUGA.1)	No	Sb03g002010	LOC_Os01g41210	CA246160 (inflorescence), DQ115076 (callus).
115J16 (132,900 bp)	Protein brittle-1 chloroplast precursor (*pb*)	Yes	Sb07g027010	LOC_Os08g40850	CA074816 (apical meristem), CA202529 (inflorescence).
	exocyst complex subunit family protein (exo)	Yes	Sb07g027000	LOC_Os08g40840	No transcript.
	Pumilio domain-containing protein (ppd1)	No	Sb07g026995	LOC_Os08g40830	CA099599 (calli), TA38834_4547 (apical meristem, inflorescence, root tips, callus and leaf roll).
	Class I Mutator-like transposase (SC-MuI.1)	No	Absent	Absent	DQ115055 (inflorescence and rachis).
086H20 (fragment: 143,827 bp)	4-hydroxy-3-methylbut-2-enyl diphosphate reductase (hme)	No	Sb01g009140	LOC_Os03g52170	TA33519_4547 (inflorescence, root apex, stalk bark).
	Regulatory protein (*rp*)	Yes	Sb01g009150	LOC_Os03g52160	TA37774_4547 (inflorescence, seedling, leaf roll, stalk bark), TA49600_4547 (root tips, seedling).
	Putative leishmanolysin-like protein (*l*)	No	Sb01g009170	LOC_Os03g52150	CA177995 (first apical stalk), TA33915_4547 (shoot-root transition zone, inflorescence and rachis), TA33917_4547 (inflorescence and rachis).
	Expressed protein (*ep1*)	Yes	Sb01g009180	LOC_Os03g52130	Sorghum: CD428305; Rice: AK070777.
	Class I Mutator-like transposase (SC-MuI.2)	No	Absent	Absent	DQ115055 (inflorescence and rachis).
249C12 (fragment: 75,471 bp)	Expressed protein (*ep2*)	No	Sb10g024710	LOC_Os06g42660	Rice: AK107791 (inflorescence)
	Class IV MUSTANG (SC-MUGB.1)	No	Sb10g024700	LOC_Os06g42640	TA40072_4547 (root tips, leaf roll, root apex).
	Protein B3 DNA binding domain containing protein (b3)	Yes	Unknown*	LOC_Os06g42630	No transcript.
	Hypothetical protein (hp)	No	Sb10g024690	LOC_Os06g42620	No transcript.
007O13 (fragment: 14,093 bp)	Class II Mutator-like transposase (SC-MuII.1)	No	Absent	Absent	TA46073_4547 (apical meristem).

*b3 was identified in sorghum even when is not annotated at Phytozome (http://www.phytozome.net/sorghum)

1: the gene name abbreviation is between parenthesis

2: presence of frame shifts or stop codons compared with orthologous protein sequence of sorghum

3: sorghum locus identification according to Phytozome (http://www.phytozome.net/sorghum)

4: rice locus identification according to TIGR (http://www.tigr.org/tdb/e2k1/osa1)

5: identified transcript in Plant Transcript Assemblies fhttp://plantta.tigr.orgD. the tissue is indicated in parenthesis. All transcripts belong to sugarcane except when indicated

6: a fragment of the BAC clone was sequenced and annotated

### Mutator System in Sugarcane

**Fig. 1:**
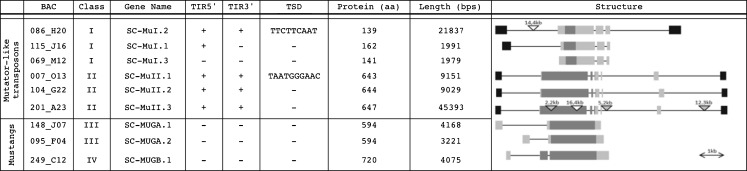
Genomic structure of the four Mutator-like classes found in sugarcane sequenced BACs. Schematic representation of the genomic features of Class I (ScMuI) and Class II (ScMuII) transposon units, and Class III (*MUGA*) and Class IV (*MUGB*) MUSTANG genes isolated from sugarcane R570 BAC clones. *Light grey boxes* represent exons drawn to scale. *Dark grey boxes* correspond to portions with similarity to a Mutator transposase protein domain. *Black boxes* represent terminal inverted repeats (TIRs) bordering transposon units. TSD; are the target site duplication sequence identified at the immediate border of the transposon adjacent to the TIR. (*plus sign*/*minus sign*) denotes presence or absence of the featured region of the transposon in the sequenced BAC. *Triangles* represent LTR retrotransposons inserted inside sequenced elements

All six BAC clones sequenced corresponded to a Mutator system containing loci that validated our selection strategy and protocol for estimating copy number. There were three MUSTANG genes: two allelic versions in the same locus of Class III, MUGA, and one Class IV, MUGB. Five transposon units were identified with the transposase domain and predicted TIRs. Two transposons belonged to Class I (SC-MuI.1 and SC-MuI.2), and three transposons to Class II (SC-MuII.1, SC-MuII.2 and SC-MuII.3). Both classes of transposons displayed distinct gene structure (Fig. 1). SC-Mul.1 and SC-Mul.2 share a common structural organization with three exons, two introns, and a partial alignment with the MuDR transposase domain. SC-Mul.2 (in BAC086H20) was characterized as a transduplicated-MULE containing a non-classified LTR retrotransposon of 14,491 bp, putative TIRs with 88 % identity and perfect target-site duplications (TSD) of 9 bases. Only one TIR was identified for SC-Mul.1 (BAC115J16). Class I transposons shared 92 % similarity along their predicted proteins (Figure 1S).

Class II elements, represented by SC-MulI.1 (BAC007O13), SC-MulI.2 (BAC104G22) and SC-MulI.3 (BAC201A23) displayed a different organization consisting of five exons, four introns with an extended non-coding 3’ region. The homology along the MuDR transposase domain is longer than the one observed for Class I in that it spans exon 1 and most of exon 2. Putative TIRs identity was 98 % and TSD of 10 bp (Figure 1S). Nucleotide sequence alignment of exons, introns, and TIRs from the two Class I and single Class II elements displayed high levels of identity suggesting that elements within a Class may be components of a transposon family as was defined genetically by McClintock as interacting autonomous and non-autonomous elements.

Both Class III MUSTANG genes have a conserved gene model when compared to the corresponding sorghum locus (Sb03g002010). The predicted peptide (594 aa) exhibits a similarity of 98 % between sugarcane haplotypes and has 97 % and 85 % similarity when compared to sorghum and rice, respectively. Based on the sugarcane MUSTANGs size and peptide prediction, the homologous rice locus (LOC_Os01g41210) is proposed to be 1,830 nt with a protein of 555 aa which is smaller than the values published in TIGR database (2,689nt and 689 aa). As expected for non-TEs no TIRs and TSD were identified bordering these genes (Fig. 1).

Class IV MUSTANG from sugarcane consists of two exons, two introns, and a predicted coding region of 1,843 bp. The predicted protein has 720 aa with a similarity of 98 % and 83 % compared to sorghum (Sb10g024700) and rice (LOC_Os06g42640), respectively. Similar predicted gene structure is proposed for the rice locus but not for sorghum. As in the case of Class III MUSTANG, no TIR or TSD were evident (Fig. 1).

### Mutator Class II In Situ Hybridization

**Fig. 2:**
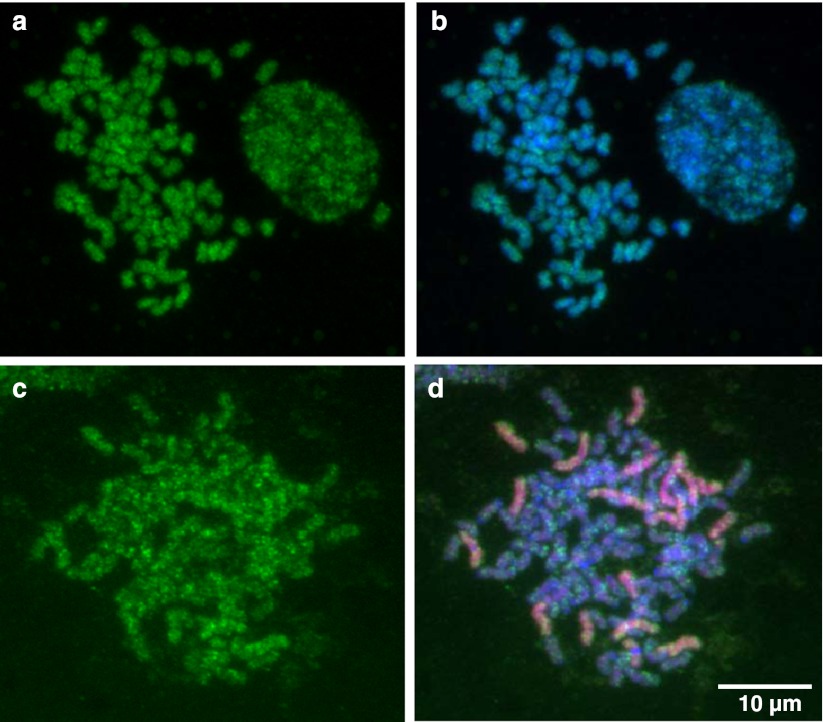
Distribution of Mutator class II on chromosomes. In situ hybridization of the Class II Mutator-like transposase clone TE109, detected by FITC fluorescence (*green*) to metaphase chromosomes of sugarcane ROC10 cultivar (**a** and **b**) and a hybrid *Saccharum* x *Miscanthus* (**c** and **d**). Chromosomes are counterstained with DAPI (*blue* signal, **b** and **d**). Genomic DNA from *Miscanthus* was labeled with biotin and detected by Texas Red fluorescence (**d**)

We performed in situ hybridization to gain an understanding of the chromosomal distribution of the most abundant Mutator-like element in the sugarcane genome. Class II specific probe (TE109) was used on metaphase spreads cells from roots from the modern cultivar ROC 10 and an inter-generic (*Miscanthus* x *Saccharum*) hybrid of closely related species within the Saccharineae group. Fluorescent in situ hybridization suggest that Class II Mutator-like elements are present at high copy number in most or all chromosomes and no particular distribution can be discerned on *Saccharum* chromosome arms (Fig. 2a, b panel) or in *Miscanthus* (Fig. 2c, d panel). These FISH experiments validate previous genome hybridization analyses reporting high copy numbers of this element (Saccaro et al. 2007) that permits depicting its pattern of distribution in sugarcane and *Miscanthus* chromosomes. SC-MulI belongs to a lineage that transposed actively in both Saccharineae and rice genomes.

### Gene Composition and Synteny Conservation with Sorghum and Rice

We identified 12 genes from BAC sequencing based on sequence alignment with sorghum. The corresponding orthologous regions from rice and sorghum revealed that gene order and content are conserved between these species along the five sugarcane loci (Table 1). MUGA and MUGB are located on syntenic regions adding support to an early domestication event. Nine of these predicted genes have corresponding transcripts in sugarcane transcript assemblies, two have transcripts detected in either rice or sorghum, and three have no transcript detected (Table 1). Three genes corresponding to syntenic regions of sorghum chromosome 3 and rice chromosome 1 were identified simultaneously in two R570 BACs. The three genes are: (1) a putative DEAH (Asp-Glu-Ala-His) box polypeptide 35 (DEAD-like helicases superfamily) similar to ATP-dependent RNA helicase (*rnah*), (2) a calcium-binding protein (*cbp*), and (3) a Class III MUSTANG (MUGA). Conservation of gene order and gene content plus the existence of shared retrotransposon insertions suggest these two BACs correspond, most probably, to two different sugarcane haplotypes. Three additional genes were identified with orthologous regions in sorghum chromosome 7 and rice chromosome 8: (1) a protein brittle-1 chloroplast precursor (*pb*), (2) an exo70 exocyst complex subunit family protein (*exo*), and (3) a pumilio domain-containing protein (*ppd1*). A 4-hydroxy-3-methylbut-2-enyl diphosphate reductase (*hme*), a regulatory protein (*rp*), a putative leishmanolysin-like protein (*l*), and an expressed protein (ep1) were identified as orthologous to genes located in sorghum chromosome 1 and rice chromosome 3. Finally, orthologous genes located in sorghum chromosome 10 and rice chromosome 6 include a hypothetical protein (*hp*), a protein B3 DNA binding domain containing protein (*b3*), a Class IV MUSTANG (MUGB), and an expressed protein (*ep2*) (Table 1). Except for the insertion of SC-MulI.1, no gene was identified in BAC 007O13 sequence probably due to the small size of contig assembly.

Gene exon and intron content was compared to rice and sorghum orthologous regions and found to be conserved for all genes identified in this study (Table 2). Intron size variation is a common among these species. Conversely, exon size is mostly conserved, as is the protein length with the exception of *ppd1* which is quite large in rice.

**Table 2:** Gene features predicted

Gene^1^	Number of exons^2^	Total length of exons (bp)				Number of introns^2^	Total length of introns (bp)				Total length of protein (aa)			
		*Sc*	*Sc* ^*5*^	sorghum	rice		*Sc*	*Sc* ^5^	sorghum	rice	*Sc*	*Sc* ^5^	sorghum	rice
*rnah*	23	2,103	1,726^4^	2,103	2,103	22	12,366	11,032	10,037	9,561	700	574^4^	700	700
*cbp*	7	1,722	1,692	1,725	1,755	6	1,890	2,085	1,927	2,912	573	563	574	584
*MUGA*	1	1,785	1,785	1,785	1,830	1	729	1,756	1,058	–	594	594	594	555
*pb*	5	1,234		1,149	1,158	4	8,477		3,288	2,776	410		382	385
*exo*	2	2,065		2,082	2,064	1	118		105	531	687		693	687
*ppd1*	9	2,295		2,292	5421	8	6,154		2,778	7,654	764		763	1,806
*hme*	10	1,404		1,395	1,380	9	2,061		2,354	1,372	467		464	459
*rp*	3	1,048		1,047	1,056	2	205		251	437	348		348	351
*l*	16	2,502		2,502	2,538	15	5,225		11,413	6,229	833		833	845
*ep1*	3	362		366	390	2	959		731	954	119		121	129
*ep2*	1	324		363	387	–	–		–	–	107		120	128
*MUGB*	3	2,163		2,163	2,169	2	1,220		73	70	720		720	722
*b3*	7	1,187		1,187	1,182	6	2,194		2,194	2,058	394		394	393
*hp*	3	204		189	408	2				444	67		62	135

1: Gene name abbreviation from Table 1

2: Number of exons and introns from the species with the most number

3: *Sc.* sugarcane

4: Truncated BAC end

5: haplotypes variant

Sorghum and rice orthologues appear to be functional genes present as full length ORFs with no frame shifts or stop codons. However, based on the presence of either stop codons or frame shifts (Table 1) sugarcane sequenced haplotypes corresponding to *pb*, *exo*, *rp*, *ep1*, and *b3* genes appear to be pseudogenes.

In an attempt to compare the evolution of these gene regions, the degree of conservation for each identified gene was evaluated by calculating the rate of synonymous and non-synonymous substitution (*ds*/*dn*). For all 14 genes analyzed *ds* is always higher than *dn,* but with different ratio values (Figure 2S). Test of selection were performed to assess whether genes were undergoing neutral (*ds* = *dn*) or purifying selection (*ds* > *dn*). The four genes *hp*, *ep2*, *b3* and *rp* with a ratio ds/dn below 3 denoted neutral selection (*p* < 0.05) while the rest of the genes (*rnah*, *cbp*, *MUGA*, *MUGB*, *ep1*, l, *hme*, *pb*, *exo* and *ppd1*) revealed a purifying selection. No correlation was observed between the ds/dn rate and the presence of a stop codon or frame shift or any identified transcript (Table 1). This result suggests that these mutations are very recent events.

The two classes of sugarcane MUSTANG genes (*MUGA* and *MUGB*) display *ds* > *dn*, as the orthologues identified in rice and sorghum, probably denote that the locus and sequenced alleles are under functional selection. However, their genomic environment presents distinct selective pressures. The genomic region of gene *MUGA*, (BACs 095F04 and 148J07) was the only one that displayed homogeneous selective constraint for all the identified genes, none of which had stop codons or frame shifts. The genes localized close to *MUGA* are highly conserved (*rnah* and *cbp*), despite the fact that in rice these genes are distantly positioned (17.5 Mb) on chromosome 1, while genes in the region of *MUGB* harbor lower *ds*/*dn* values supporting neutral selection (Figure 2S). Collectively, these results suggest that the sequenced haplotype spanning *MUGA* contains all functional genes; conversely, *MUGB* is the only active gene within the corresponding sequenced haplotype. Supporting evidence is the fact that *b3* gene in *MUGB* genomic region, harbors an in frame stop codon.

### MUSTANG Class III Gene is Located at an Orthologous Region

**Fig. 3:**
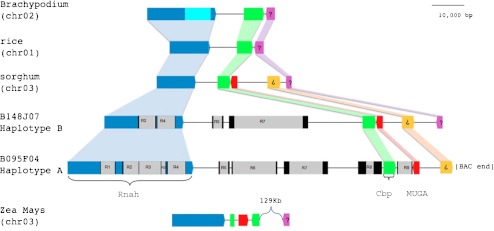
Gene order is conserved at Class III MUSTANG locus in Poaceae. Schematic representation of orthologous region from *Brachypodium*, rice, sorghum, maize, sugarcane haplotype **a** and sugarcane haplotype **b**. Exons are indicated as *colored boxes*. Connectors mark orthologous genes. *Grey boxes* are inserted retrotransposons with their corresponding LTRs shown as *black boxes*. Gene names are indicated on the sugarcane haplotype **a**

Since Mutator-like transposase sequences belonging to Class III are considered domesticated transposases with low copy number (Saccaro et al. 2007), selection of BACs representing two haplotypes of the same locus was made possible. Restriction profile and hybridization experiments (data not shown) suggest that haplotype A (BAC095F04) could correspond to that of the *S. officinarum* progenitor while haplotype B (BAC148J07) could correspond to that of the *S. spontaneum* parent, however, the analysis solely based on the restriction pattern was not conclusive. These results presented a unique opportunity to compare the orthologous genomic regions among five grass species. Figure 3 presents a graphical view of the genomic region showing incremental changes in DNA content of both sugarcane haplotypes compared to sorghum, *Brachypodium,* and rice. The maize inbred line B73 corresponding region in chromosome 3 seems to be rearranged. Recent reports have highlighted the extensive colinearity existing in Poaceae species (Bennetzen and Ramakrishna 2002; Jannoo et al. 2007; Paterson et al. 2009). The two sugarcane haplotypes showed more intense retrotransposon activity than did the other species. A total of nine insertions were identified of which four are shared between the two sugarcane haplotypes (R2, R4, R5 and R7). The remaining insertions are unique to haplotype A (*S. officinarum*). The LTR nucleotide identity of the shared retrotransposon insertions is: 99 % for R2, 99 % for R4, 98 % for R5 and 96 % for R7.

### Repetitive Elements

Of 579,352 sequenced bases, 198,038 correspond to repetitive DNA mainly composed of retrotransposons. A total of 19 full-length elements (156,031 bases) were identified harboring both LTRs and internal coding sequences. Of these, one element of 4,848 bases belongs to a Ty1/copia lineage and three totaling 19,841 bases correspond to a Ty3/gypsy superfamily. The remaining elements were not assigned to any particular family. Most of the insertions were found in intergenic regions and 44,068 bases were found to be located within introns. None of the elements were disrupting coding regions. The relative abundance of genes and TEs along the distinct genomic regions analyzed was measured over a genomic window flanked by orthologous genes, making it possible to compare four different regions. In all cases, the sugarcane genomic fragments were larger. This difference, at least in part, was caused by the presence of retrotransposons that were absent in the rice and sorghum orthologous regions. Retrotransposon abundance was measured in each genomic string and varied from 25 % to 63 % of the sequenced bases in sugarcane, which support the concept that specific retrotransposon amplification may correlate with genome size amplification, or conversely, to the corresponding elimination from rice and sorghum genomes (data not shown). No solo-LTRs were found in these sequenced BACs.

## Discussion

The sugarcane Mutator system is composed of four transposase lineages of which the structures of 9 genomic copies are described in the present work. Classes I and II comprise true transposon units that carry not only the transposase domain but also terminal inverted repeats. Also, duplicated insertion sites were identified for a couple of insertions. One of the Class I copies exemplifies transduplicated elements as previously described in rice (Jiang et al. 2004; Saccaro et al. 2007). Class III and IV are proposed to be domesticated transposase lineages and do not harbor TIRs. Supporting their domesticated nature is the fact that they are located in syntenic regions across rice, sorghum and sugarcane genomes. These are considered cellular genes as previously described in Arabidopsis and rice (Cowan et al. 2005; Lin et al. 2008). On the other hand, none of the Class I and II copies sequenced in this work are present in conserved regions which suggests recent activity. FISH analysis confirmed earlier predictions that Class II Mutator-like elements are highly represented in the sugarcane genome (Saccaro et al. 2007). Similar trends can be proposed in the case of *Miscanthus*, as the *Miscanthus* chromosomes clone displayed hybridizing signals comparable to those on *Saccharum* chromosomes. Therefore the results presented here disclose that each Mutator-like transposase class analyzed has a particular molecular structure suggesting lineage specific evolution from ancient ancestors of the Saccharinae lineage.

Recently, Jannoo et al. (2007) analyzed the orthologous Adh1 genomic regions from *S. officinarum* and *S. spontaneum* sugarcane haplotypes, and sorghum, to estimate that the two *Saccharum* species diverged from sorghum 1.5-2 MYA and 8-9 MYA, respectively. Those authors also proposed that the high polyploidy in sugarcane did not induce a generalized genome reshaping. The results presented here are in agreement with those earlier reports indicating that, irrespective of retrotransposon amplification, colinearity is kept in these analyzed regions. Based on the observation that the *Adh1* gene from several Poaceae sits on the chromosome surrounded by LTR-retrotransposons, Bennetzen and Ramakrishna (2002) proposed that plants have evolved effective insulators to protect genes from non-genic regions. Jannoo et al. (2007) extended the *Adh*1 region comparative study in sugarcane to confirm the higher content of LTR-retrotransposons in sugarcane genome. Garsmeur et al. (2011) described the structure of eight BACs corresponding to seven haplotypes in the *Bru*1 locus. In addition, the present work characterized several sugarcane genomic loci containing Mutator-like transposases, with more than half a million of sequenced bases.

All sequenced loci in the present work have a corresponding syntenic region in sorghum and rice. Microcolinearity was interrupted by novel LTR retrotransposon insertions, even when the sugarcane haplotypes were compared extending the observation made at *Adh*1 (Jannoo et al. 2007) and *Bru*1 (Garsmeur et al. 2011) loci. Wang et al. (2010) analyzed 20 sugarcane BAC clones selected using euchromatic sorghum probes and found that 53 % of sugarcane BACs aligned with sorghum. The unaligned region contained non-coding and repetitive sequences, thus confirming colinearity in the genic regions between sugarcane and sorghum. Each locus analyzed in the current work has a unique genome amplification pattern compared to sorghum, mostly associated with LTR-retrotransposons. These several examples, together with the ones presented, suggest that different regions have different dynamics in genome expansion and contraction. Fu and Dooner (2002) also detected extensive retrotransposon activity underlying maize haplotype variation when comparing two haplotypes, one from B73 and the other from McC inbred lines. Bennetzen and Ramakrishna (2002) put forward the notion that the domestication process from wild to cultivated plant species may result in rapid loss of genes necessary to wild populations and that hybrid vigor and yield performance may correspond to intrinsic outcomes of chromatin biochemistry not yet understood.

Genome dynamics varies greatly between organisms contributing to different genomes sizes. Two mechanisms have been uncovered as the most important in genome obesity: polyploidization and TEs amplification (Wendel 2000; Bennetzen 2000; Bennetzen 2002). Comparative genomics offer numerous opportunities for advancing and understanding genome size evolution, and are more revealing when phylogenetically close species are compared.

Gene conservation among sugarcane was diverse. The presence of five pseudogenes in sugarcane sequences denotes that some haplotypes in the polyploid hybrid are probably not functional. The helicase–rnah (that presents no stops or frame shifts), displays a *ds*/*dn* of 3 between the two haplotypes while between sugarcane and sorghum the values are 13 (with haplotype A) and 16 (with haplotype B). Our results, as well as Adh region previously reported (Jannoo et al. 2007), reveal that TE content over orthologous genomic intervals in both, gene-rich and non-coding regions seems higher in *S. officinarum*. Most genes identified possess transcripts thus supporting the concept that the genes identified in these sequenced BACs correspond to functional loci. However, we stress that it is not possible at this point to determine if these are expressing alleles.

*S. officinarum* and *S. spontaneum* display perfect gene colinearity with related species, in contrast to previous reports describing the massive rearrangements in maize (Ma et al. 2005) and wheat (Chantret et al. 2005). The four LTR-retrotransposons, that are not present in the related species, the R2 and R4 retrotransposons inserted in the helicase intron, and the R5 and R7 that we detected in the inter-genic region, might have occurred before the *Saccharum* species divergence. Furthermore, retrotransposon insertion profiles can distinguish the two sequenced haplotypes thus supporting their use as allelic markers. In this case, their insertions might have occurred during species divergence or as a product of the inter-specific hybridization, like the very recent insertion of R1. Since this inter-specific cross was made only a century ago, sugarcane hybrid cultivars might be in the process of initiating genome remodeling and undergoing TE amplification as proposed by Soltis and Soltis (2000) for other angiosperms species. Since 2.3 % of the sugarcane transcriptome is composed of TEs this could be an indication of a first activation step (Araújo et al. 2005). The amount of retrotransposons found along all five genomic regions studied in this work is higher in the sugarcane hybrid genome than in related species. This evidence suggests that modern sugarcane hybrids are initiating TE amplification. It would be of interest to explore this possibility in *S. officinarum* and *S. spontaneum* at the species level. Further studies may provide knowledge to advance of genomic haplotype variation and to explain the forces driving dissimilar evolutionary rates on neighboring gene.

As observed by Jannoo et al. (2007) for the *Adh*1 locus, sugarcane chromosomal regions present higher content of retrotransposons, as revealed by both density and percentage of retrotransposons, compared to rice and sorghum. The exhaustive annotation and analysis of the produced sequences disclosed that each Mutator transposase class displays a particular molecular structure, supporting lineage specific evolution. Moreover, the results showed that these elements are ancient inhabitants in the Saccharinae lineage. MUSTANG genes are located in syntenic regions across grasses and posses the same gene structure as in rice and sorghum, which would be expected for a host functional gene. Retrotransposon insertions that discriminate sugarcane haplotypes and amplification were detected in all members of the genus Saccharum.

Results from the present work extend knowledge on the Mutator system from Poaceae by providing molecular structure information and genomic distribution pattern from four lineages previously identified from sugarcane transcriptome.

## Materials and Methods

### BAC Selection and Sequencing

Twelve clones for Classes I, II and IV, and the six identified clones for Class III were fingerprinted digesting 1 Pg of BAC DNA with *Hind*III (Table S1) previously identified from high-density filters of a BAC library for sugarcane R570 cultivar (Tomkins et al. 1999) and class-specific probes, Saccaro et al. (2007). An aliquot of 10 ng of *Hind*III digested BAC DNA was run in parallel to 15 pg of *S. officinarum* and *S. spontaneum* DNA, also restricted with HindIII, to compare hybridization profiles with the class-specific probes. Fresh sugarcane leaf tissue of *S. officinarum* (clone Badila) and *S. spontaneum* (clone Mandalay) species were kindly provided by Dr. Eugênio Ulian from the Centro de Tecnologia Canavieira–CTC (Piracicaba, São Paulo, Brazil). DNA isolation was performed according to Doyle and Doyle (1987). Gel blotting was done according to Grivet et al. (1996).

DNA of the six selected BAC clones was extracted according to Sambrook et al. (1989). Aliquots of 10 pg were mechanically sheared by sonication, end repaired, and cloned into blunt ended pMOSBlue vetor according to manufacturer recommendations (pMOSBlue Blunt Ended Cloning Kit, RPN5110, Amersham Biosciences). Shotgun clones were end sequenced with universal vector primers on an ABI 3130 DNA sequencer using Big Dye Terminator v3.1 Cycle Sequencing Kit (Applied Biosystems, Inc). Reads assembly was performed with Phred + Phrap + Consed package, version 14.00 (Gordon et al. 1998). When necessary, primers were constructed for gap filling. Quality of the consensus nucleotide sequence was set at 1 error accepted /10,000 bases. (or, until all bases reached phred quality t20).

### Sequence Annotation

Annotation was based on sequence comparison with Sorghum bicolor genomic sequence (Phytozome database http://www.phytozome.net/sorghum). Rice orthologous sequences were identified in TIGR (http://www.tigr.org/tdb/e2k1/osa1/). A gene was considered full length when the sugarcane locus encompasses the full length sorghum coding sequence (CDS). TEs were determined by a combined approach based on BLASTN against the previously full length cDNA sequenced in-house GaTE database (Araújo et al. 2005) and Repeat Masker (http://www.repeatmasker.org/). Retrotransposons were identified using BLASTX against TIGR data base (Altschul et al. 1990), while LTRs were determined with LTR finder (Xu and Wang 2007) and BLAST 2 Sequences (Tatusova and Madden 1999). For both, genes and TEs, between 80 and 100 % coverage over the transcribed region was mandatory and a cut off value of E-10 was set as criteria to nominate a predicted gene name or a specific TE lineage. Sugarcane sequences have been deposited in NCBI under accession numbers: GU080318, GU080319, GU080320, GU080321, GU080322, GU080323

### Sequence Divergence Analyses

Alignment of coding regions were performed with CLUSTAL W multiple-alignment (version 1.5) (Thompson et al. 1994) and manually adjusted with reference to aa alignment. Deletions, whenever present, were also removed. Synonymous (*ds*) and non-synonymous (*dn*) distances and its standard errors were calculated with MEGA 3.1 (Kumar et al. 2004) using Nei-Gojobori method (Jukes-Cantor). Codon bias was determined by Nc value computed using CodonW (http://bioweb.pasteur.fr/seqanal/interfaces/cadonw.html).

Test of selective constraints was also performed with MEGA 3.1 (Kumar et al. 2004) using Nei-Gojobori method. To reject the null hypothesis of neutral selection (*ds* = *dn*) a *p* < 0.05 in the Z-test was considered.

Relative rate test was performed with HYPH (http://www.hyph.org) using codon model. To reject the homogeneity null hypotheses a *p* < 0.05 in the F2 test was considered.

### Fluorescent In Situ Hybridization

Chromosomes from sugarcane cultivar ROC10 were prepared as described in D’Hont et al. (1996). The slides were stored at −80 °C until use. Slides were treated with RNAse (1 pg/ml) at 37 °C for 45 min, denatured for 3 min. in 70 % formamide in 2XSSC at 80 °C, then dehydrated through an ethanol series at −20 °C. The hybridization mixture (30 Pl per slide) consisted of 50 % formamide, 10 % dextran sulphate, 2XSSC, 1 % SDS and the DNA probe. The probe was prepared from 1Pg of DNA minipreparation from TE109, a Class II Mutator-like element (Rossi et al. 2004) labelled with digoxigenin-11-dUTP (Roche) by nicktranslation, using the Nick Translation Mix (Roche) and 1 μg of *Miscanthus* genomic DNA labeled with biotin- 14-dCTP by nick-translation, using the BioPrime DNA labeling System (Invitrogen). The hybridization mixture was denatured for 10 min in boiling water. Hybridization was performed overnight in a moist chamber at 37 °C. The washes, the detection of digoxigenin with FITC and the biotin with Texas Red, the signal amplification were performed as described in D’Hont et al. (1996). The slides were mounted in Vectashield antifade solution with DAPI as counterstaining (Vector Lab.) and captured in a CCD video camera Sensys Photometrix. Digital photographs were taken using QFISH software (Leica).

## Electronic Supplementary Material

Below is the link to the electronic supplementary material.

Table S1 R570 BAC library clones confirmed to have a Mutator-like element from each of the described classes in Rossi et al. 2004. (DOC 68 kb)

ESM 1 (PPT 409 kb)
