# Supplementary material for: Mutator System Derivatives Isolated from Sugarcane Genome Sequence
Source: Trop Plant Biol. 2012 Jul 6;5(3):233–43. doi: 10.1007/s12042-012-9104-y (PMC3418495; doi:10.1007/s12042-012-9104-y)
Supplement: Supplementary file 1 — R570 BAC library clones confirmed to have a Mutator-like element from each of the described classes in Rossi et al. 2004. (DOC 68 kb) [file 12042_2012_9104_MOESM1_ESM.doc]

| **Table S1.** R570 BAC library clones confirmed to have a Mutator-like element from each of the described classes in Rossi et al., 2004. | |
| --- | --- |
|  |  |
| Mutator-like Transposase Class | BAC clone identifier R570 genomic library |
|  |  |
| Class I | 014P10 |
|  |  |
| Class I | 037N09 |
|  |  |
| **Class I** | **086H20** |
|  |  |
| Class I | 069M12 |
|  |  |
| Class I | 102G20 |
|  |  |
| **Class I** | **115J16** |
|  |  |
| Class I | 165I15 |
|  |  |
| Class I | 173L07 |
|  |  |
| Class I | 231N16 |
|  |  |
| Class I | 238K02 |
|  |  |
| Class I | 258H24 |
|  |  |
| Class I | 255G12 |
|  |  |
| Class II | 002J11 |
|  |  |
| **Class II** | **007O13** |
|  |  |
| Class II | 074O01 |
|  |  |
| Class II | 082J17 |
|  |  |
| Class II | 104G22 |
|  |  |
| Class II | 137B14 |
|  |  |
| Class II | 168J15 |
|  |  |
| Class II | 172P01 |
|  |  |
| **Class II** | **201A23** |
|  |  |
| Class II | 239C20 |
|  |  |
| Class II | 260B03 |
|  |  |
| Class II | 248D02 |
|  |  |
| Class III | 048I02 |
|  |  |
| **Class III** | **095F04** |
|  |  |
| Class III | 104es18 |
|  |  |
| Class III | 125P17 |
|  |  |
| **Class III** | **148J07** |
|  |  |
| Class III | 230L02 |
|  |  |
| Class IV | 045G21 |
|  |  |
| Class IV | 043B20 |
|  |  |
| Class IV | 093D13 |
|  |  |
| Class IV | 085K02 |
|  |  |
| Class IV | 132C06 |
|  |  |
| Class IV | 137O14 |
|  |  |
| Class IV | 153J08 |
|  |  |
| Class IV | 171B10 |
|  |  |
| Class IV | 214B14 |
|  |  |
| Class IV | 208F12 |
|  |  |
| **Class IV** | **249C12** |
|  |  |
| Class IV | 245N03 |
