# Supplementary material for: Mutator System Derivatives Isolated from Sugarcane Genome Sequence
Source: Trop Plant Biol. 2012 Jul 6;5(3):233–43. doi: 10.1007/s12042-012-9104-y (PMC3418495; doi:10.1007/s12042-012-9104-y)
Supplement: Supplementary file 2 — (PPT 409 kb) [file 12042_2012_9104_MOESM2_ESM.ppt]

## Slide 1
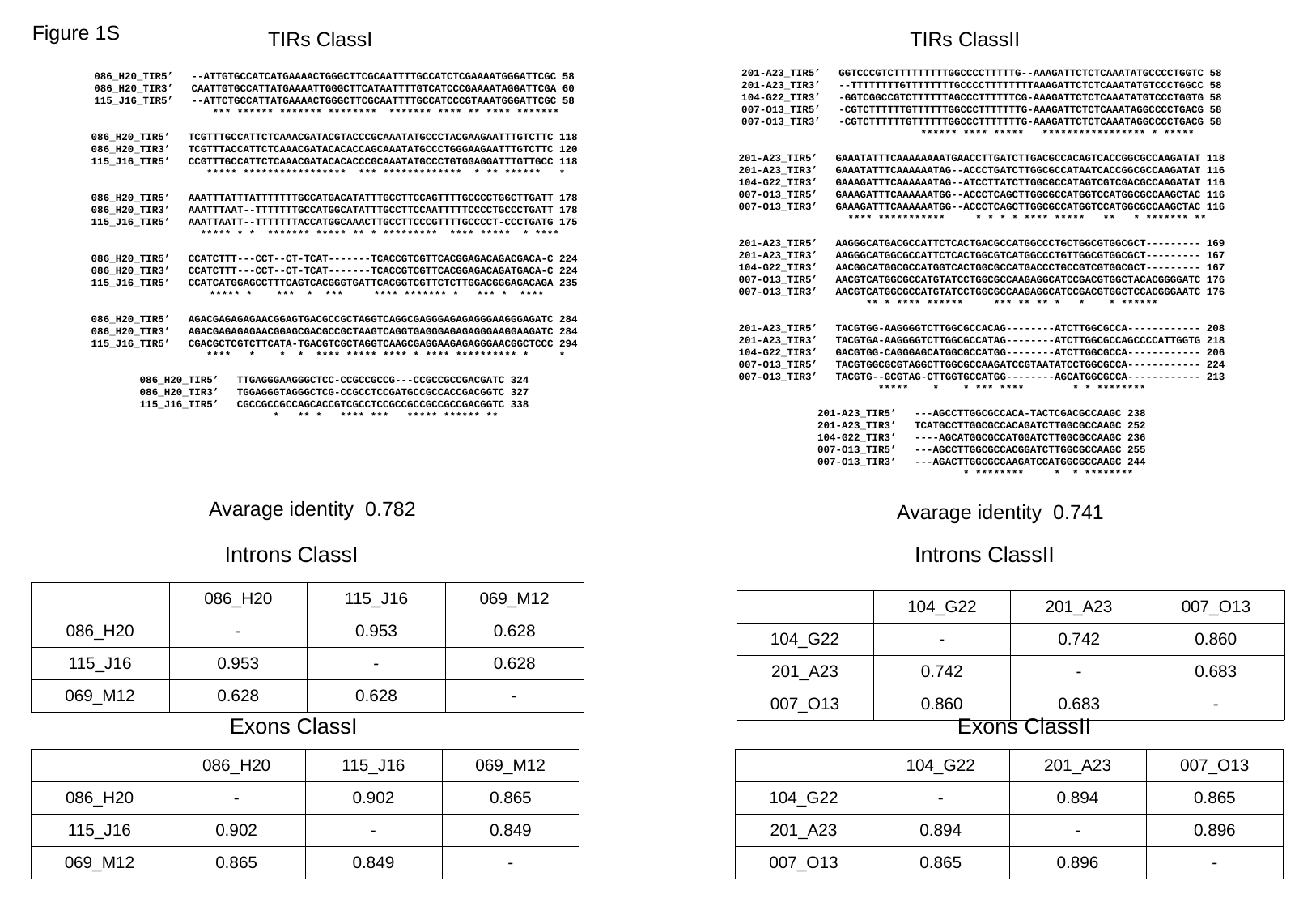

Figure 1S
TIRs ClassI
TIRs ClassII
201-A23_TIR5’ GGTCCCGTCTTTTTTTTTGGCCCCTTTTTG--AAAGATTCTCTCAAATATGCCCCTGGTC 58
201-A23_TIR3’ --TTTTTTTTGTTTTTTTTGCCCCTTTTTTTTAAAGATTCTCTCAAATATGTCCCTGGCC 58
104-G22_TIR3’ -GGTCGGCCGTCTTTTTTAGCCCTTTTTTCG-AAAGATTCTCTCAAATATGTCCCTGGTG 58
007-O13_TIR5’ -CGTCTTTTTTGTTTTTTGGCCCTTTTTTTG-AAAGATTCTCTCAAATAGGCCCCTGACG 58
007-O13_TIR3’ -CGTCTTTTTTGTTTTTTGGCCCTTTTTTTG-AAAGATTCTCTCAAATAGGCCCCTGACG 58
 ****** **** ***** ***************** * *****
201-A23_TIR5’ GAAATATTTCAAAAAAAATGAACCTTGATCTTGACGCCACAGTCACCGGCGCCAAGATAT 118
201-A23_TIR3’ GAAATATTTCAAAAAATAG--ACCCTGATCTTGGCGCCATAATCACCGGCGCCAAGATAT 116
104-G22_TIR3’ GAAAGATTTCAAAAAATAG--ATCCTTATCTTGGCGCCATAGTCGTCGACGCCAAGATAT 116
007-O13_TIR5’ GAAAGATTTCAAAAAATGG--ACCCTCAGCTTGGCGCCATGGTCCATGGCGCCAAGCTAC 116
007-O13_TIR3’ GAAAGATTTCAAAAAATGG--ACCCTCAGCTTGGCGCCATGGTCCATGGCGCCAAGCTAC 116
 **** *********** * * * * **** ***** ** * ******* **
201-A23_TIR5’ AAGGGCATGACGCCATTCTCACTGACGCCATGGCCCTGCTGGCGTGGCGCT--------- 169
201-A23_TIR3’ AAGGGCATGGCGCCATTCTCACTGGCGTCATGGCCCTGTTGGCGTGGCGCT--------- 167
104-G22_TIR3’ AACGGCATGGCGCCATGGTCACTGGCGCCATGACCCTGCCGTCGTGGCGCT--------- 167
007-O13_TIR5’ AACGTCATGGCGCCATGTATCCTGGCGCCAAGAGGCATCCGACGTGGCTACACGGGGATC 176
007-O13_TIR3’ AACGTCATGGCGCCATGTATCCTGGCGCCAAGAGGCATCCGACGTGGCTCCACGGGAATC 176
 ** * **** ****** *** ** ** * * * ******
201-A23_TIR5’ TACGTGG-AAGGGGTCTTGGCGCCACAG--------ATCTTGGCGCCA------------ 208
201-A23_TIR3’ TACGTGA-AAGGGGTCTTGGCGCCATAG--------ATCTTGGCGCCAGCCCCATTGGTG 218
104-G22_TIR3’ GACGTGG-CAGGGAGCATGGCGCCATGG--------ATCTTGGCGCCA------------ 206
007-O13_TIR5’ TACGTGGCGCGTAGGCTTGGCGCCAAGATCCGTAATATCCTGGCGCCA------------ 224
007-O13_TIR3’ TACGTG--GCGTAG-CTTGGTGCCATGG--------AGCATGGCGCCA------------ 213
 ***** * * *** **** * * ********
201-A23_TIR5’ ---AGCCTTGGCGCCACA-TACTCGACGCCAAGC 238
201-A23_TIR3’ TCATGCCTTGGCGCCACAGATCTTGGCGCCAAGC 252
104-G22_TIR3’ ----AGCATGGCGCCATGGATCTTGGCGCCAAGC 236
007-O13_TIR5’ ---AGCCTTGGCGCCACGGATCTTGGCGCCAAGC 255
007-O13_TIR3’ ---AGACTTGGCGCCAAGATCCATGGCGCCAAGC 244
 * ******** * * ********
086_H20_TIR5’ --ATTGTGCCATCATGAAAACTGGGCTTCGCAATTTTGCCATCTCGAAAATGGGATTCGC 58
086_H20_TIR3’ CAATTGTGCCATTATGAAAATTGGGCTTCATAATTTTGTCATCCCGAAAATAGGATTCGA 60
115_J16_TIR5’ --ATTCTGCCATTATGAAAACTGGGCTTCGCAATTTTGCCATCCCGTAAATGGGATTCGC 58
 *** ****** ******* ******** ******* **** ** **** *******
086_H20_TIR5’ TCGTTTGCCATTCTCAAACGATACGTACCCGCAAATATGCCCTACGAAGAATTTGTCTTC 118
086_H20_TIR3’ TCGTTTACCATTCTCAAACGATACACACCAGCAAATATGCCCTGGGAAGAATTTGTCTTC 120
115_J16_TIR5’ CCGTTTGCCATTCTCAAACGATACACACCCGCAAATATGCCCTGTGGAGGATTTGTTGCC 118
 ***** ***************** *** ************* * ** ****** *
086_H20_TIR5’ AAATTTATTTATTTTTTTGCCATGACATATTTGCCTTCCAGTTTTGCCCCTGGCTTGATT 178
086_H20_TIR3’ AAATTTAAT--TTTTTTTGCCATGGCATATTTGCCTTCCAATTTTTCCCCTGCCCTGATT 178
115_J16_TIR5’ AAATTAATT--TTTTTTTACCATGGCAAACTTGCCTTCCCGTTTTGCCCCT-CCCTGATG 175
 ***** * * ******* ***** ** * ********* **** ***** * ****
086_H20_TIR5’ CCATCTTT---CCT--CT-TCAT-------TCACCGTCGTTCACGGAGACAGACGACA-C 224
086_H20_TIR3’ CCATCTTT---CCT--CT-TCAT-------TCACCGTCGTTCACGGAGACAGATGACA-C 224
115_J16_TIR5’ CCATCATGGAGCCTTTCAGTCACGGGTGATTCACGGTCGTTCTCTTGGACGGGAGACAGA 235
 ***** * *** * *** **** ******* * *** * ****
086_H20_TIR5’ AGACGAGAGAGAACGGAGTGACGCCGCTAGGTCAGGCGAGGGAGAGAGGGAAGGGAGATC 284
086_H20_TIR3’ AGACGAGAGAGAACGGAGCGACGCCGCTAAGTCAGGTGAGGGAGAGAGGGAAGGAAGATC 284
115_J16_TIR5’ CGACGCTCGTCTTCATA-TGACGTCGCTAGGTCAAGCGAGGAAGAGAGGGAACGGCTCCC 294
 **** * * * **** ***** **** * **** ********** * *
086_H20_TIR5’ TTGAGGGAAGGGCTCC-CCGCCGCCG---CCGCCGCCGACGATC 324
086_H20_TIR3’ TGGAGGGTAGGGCTCG-CCGCCTCCGATGCCGCCACCGACGGTC 327
115_J16_TIR5’ CGCCGCCGCCAGCACCGTCGCCTCCGCCGCCGCCGCCGACGGTC 338
 * ** * **** *** ***** ****** **
Avarage identity 0.782
Avarage identity 0.741
Introns ClassI
Introns ClassII
| | 086\_H20 | 115\_J16 | 069\_M12 |
| --- | --- | --- | --- |
| 086\_H20 | - | 0.953 | 0.628 |
| 115\_J16 | 0.953 | - | 0.628 |
| 069\_M12 | 0.628 | 0.628 | - |
| | 104\_G22 | 201\_A23 | 007\_O13 |
| --- | --- | --- | --- |
| 104\_G22 | - | 0.742 | 0.860 |
| 201\_A23 | 0.742 | - | 0.683 |
| 007\_O13 | 0.860 | 0.683 | - |
Exons ClassI
Exons ClassII
| | 086\_H20 | 115\_J16 | 069\_M12 |
| --- | --- | --- | --- |
| 086\_H20 | - | 0.902 | 0.865 |
| 115\_J16 | 0.902 | - | 0.849 |
| 069\_M12 | 0.865 | 0.849 | - |
| | 104\_G22 | 201\_A23 | 007\_O13 |
| --- | --- | --- | --- |
| 104\_G22 | - | 0.894 | 0.865 |
| 201\_A23 | 0.894 | - | 0.896 |
| 007\_O13 | 0.865 | 0.896 | - |

## Slide 2
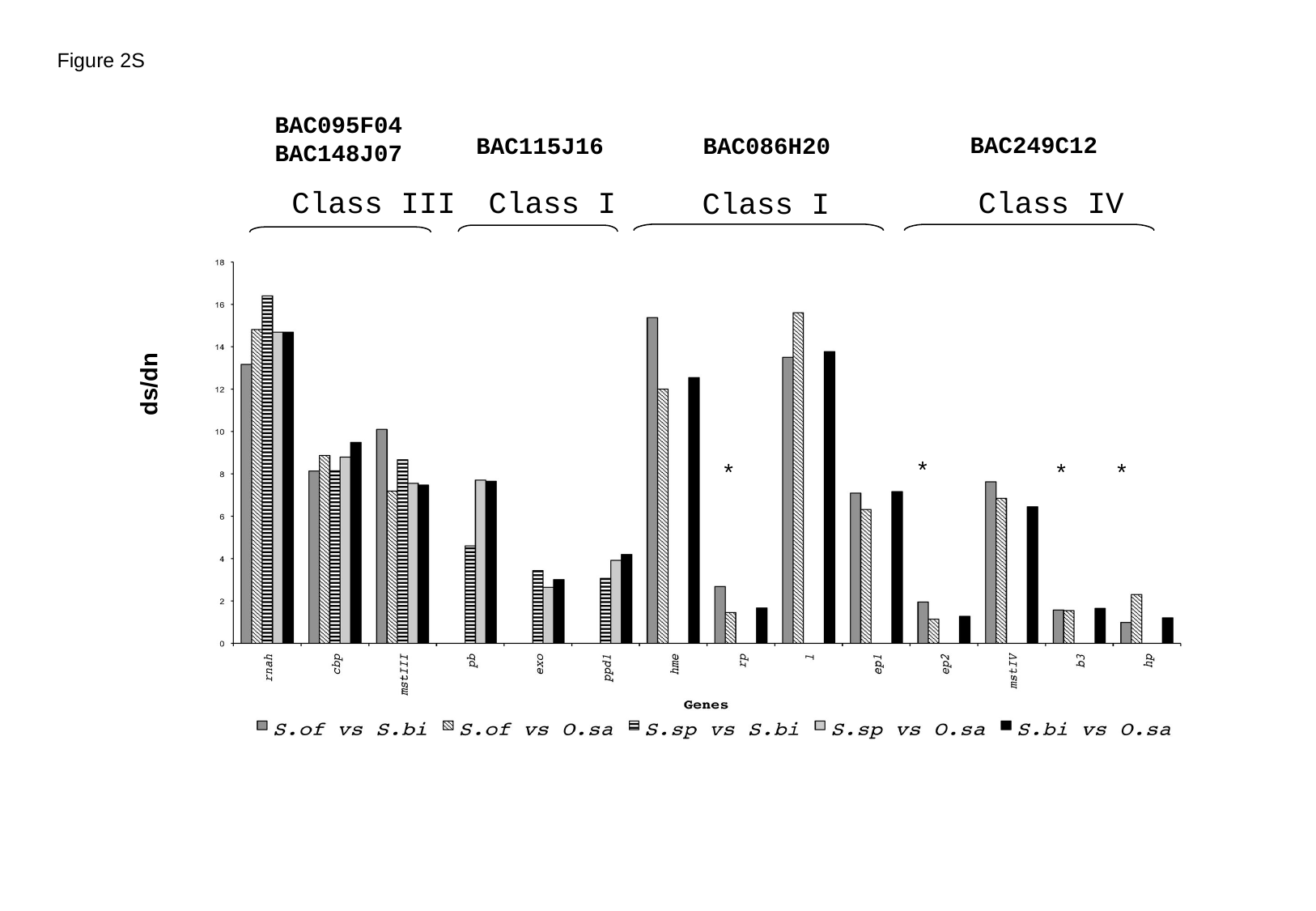

Figure 2S
BAC095F04
BAC148J07
BAC249C12
BAC115J16
BAC086H20
Class IV
Class I
Class III
Class I
ds/dn
*
*
*
*
